# Convective Heat Transfer Analysis for Aluminum Oxide (*Al*_2_*O*_3_)- and Ferro (*Fe*_3_*O*_4_)-Based Nano-Fluid over a Curved Stretching Sheet

**DOI:** 10.3390/nano12071152

**Published:** 2022-03-30

**Authors:** Asifa Ashraf, Zhiyue Zhang, Tareq Saeed, Hussan Zeb, Taj Munir

**Affiliations:** 1 Jiangsu Key Laboratory for NSLSCS, School of Mathematical Sciences, Nanjing Normal University, Nanjing 210023, China; asifa.ashraf70@yahoo.com (A.A.); zhangzhiyue@njnu.edu.cn (Z.Z.); 2 Nonlinear Analysis and Applied Mathematics (NAAM)-Research Group, Department of Mathematics, Faculty of Science, King Abdulaziz University, P.O. Box 80203, Jeddah 21589, Saudi Arabia; 3 Department of Mathematics & Statistics, Hazara University, Mansehra 21120, Pakistan; hussan_maths@hu.edu.pk; 4 Abdus Salam School of Mathematical Sciences, Government College University Lahore, Lahore 54600, Pakistan; taj.munir@sms.edu.pk

**Keywords:** *Al*_2_*O*_3_ aluminum oxide and ferro *Fe*_3_*O*_4_ nano-particles, non-linear curved sheet, convective heat transfer, velocity slip boundary condition

## Abstract

In this work, the combined effects of velocity slip and convective heat boundary conditions on a hybrid nano-fluid over a nonlinear curved stretching surface were considered. Two kinds of fluids, namely, hybrid nano-fluid and aluminum oxide ($Al_{2}O_{3}$)- and iron oxide ($Fe_{3}O_{4}$)-based nano-fluid, were also taken into account. We transformed the governing model into a nonlinear system of ordinary differential equations (ODEs). For this we used the similarity transformation method. The solution of the transformed ODE system was computed via a higher-order numerical approximation scheme known as the shooting method with the Runge–Kutta method of order four (RK-4). It is noticed that the fluid velocity was reduced for the magnetic parameter, curvature parameter, and slip parameters, while the temperature declined with higher values of the magnetic parameter, Prandtl number, and convective heat transfer. Furthermore, the physical quantities of engineering interest, i.e., the behavior of the skin fraction and the Nusselt number, are presented. These behaviors are also illustrated graphically along with the numerical values in a comparison with previous work in numerical tabular form.

## 1. Introduction and Literature Review

Boundary layer flows are characterized by Newtonian and non-Newtonian fluids. Newtonian fluids are those in which the stress is linearly proportional to strain. Examples of Newtonian fluids are mineral oil, water, gasoline, organic matter, kerosene, solvents, glycerin, alcohol, etc. The boundary layer flows of non-Newtonian fluids have attracted much attention owing to their tremendous applications in industry, manufacturing, and geothermal engineering. Examples of these applications are nuclear reactors, metallurgical processes, the spinning of fibers, casting, liquid metals space technology, crystal growth, and many more. The models of non-Newtonian fluids cannot be expressed in a single relationship because of their mutual aspects. Crane et al. [1] determined the boundary layer flow over exponentially and linear stretching surfaces. Then, Vleggaar [2] explored the laminar flow of the boundary layer on a continuous and accelerating stretching surface. The similarity analysis for the Navier–Stokes equation over the stretching surface was studied by Wang [3]. The time-dependent boundary layer flow of viscous fluid over a stretching curved surface was investigated by Sajid et al. [4].

The time-dependent boundary layer flow over a permeable curved shrinking/stretching surface was demonstrated by Rosca et al. [5]. The properties of homogeneous and heterogeneous reactions on boundary layer flow over a stretchable curved sheet were studied by Saif et al. [6]. The temperature-dependent conductivity effects on boundary layer fluid and heat transfer over a curved stretchable sheet were reported by Murtaza et al. [7]. A non-Newtonian fluid over an exponential curved stretching surface with the magnetic field was presented by by Shi et al. [8]. The computational solution of an electrically conducting macropoler fluid over a curved stretching sheet was analyzed by Naveed et al. [9].

Nano-fluids can be defined as the suspension of particles having one or more metal or non-metal nano-particles such as ($Ag,Cu,Fe,S_{1}O_{2},CuO,Al_{2}O_{3},Hg$) that are nano-sized (1–100 nm). Initially, nano-fluid improves the thermo-physical properties of nano-fluid, as was presented by Choi [10]. He concluded that thermo-physical properties promote the nano-fluid through thermal conductivity, thermal diffusivity, the volumetric fraction of the nano-particles, convective heat, and viscosity as compared to the base fluid. Furthermore, many authors have presented the characteristics of nano-particles in different fluid models, which can be seen in [11,12,13,14,15,16]. Hybrid nano-fluid along with ($Cu+Fe_{3}O_{4}/H_{2}O$) and $Cu/H_{2}O$ was initially presented by Suresh et al. [17]. Then, Shoaib et al. [18] presented numerical approaches for the MHD flow of hybrid nano-fluids with heat transfer over a moving surface.

Then, Devi et al. [19] also investigated the MHD flow of hybrid nano-fluid $Cu/Al_{2}O_{3}$ water due to a stretching surface. Hassan et al. [20] presented the characteristics of a Cu−Ag/–water hybrid nano-fluid with convective heat transfer using an inverted cone. The numerical solution for the stagnation point flow of hybrid nano-fluid (Cu−Ag/–water) was presented by Dinarvand et al. [21]. The Darcy–Forchheimer flow of hybrid nano-liquid (MWCNTs $+Fe_{3}O_{4}$/water and $SWCNTs+Fe_{3}O_{4}$/water) over a curved surface was investigated by Saeed et al. [22]. In a porous space, the viscous fluid was expressed by Darcy–Forchheimer. A mixed convection hybrid nano-fluid over a curved sheet was illustrated by Gohar et al. [23]. Waini et al. [24] explored the effects of thermal radiation on the MHD flow of a hybrid nano-fluid by considering a permeable stretching wedge. The stability analysis of the stagnation point flow of a hybrid nano-fluid with heat transfer analysis due to a shrinking sheet was computed by [25].

The effects of metallic nano-particles on the MHD flow of micropolar fluid through a vertical artery with a six-type stenosis was presented by Ashfaq et al. [26]. Yousefi et al. [27] found the analytical solution for the time-dependent stagnation point flow of a hybrid nano-fluid by considering a moving cylinder. Furthermore, the researchers presenting hybrid nano-fluid models can be seen in [28,29,30,31,32,33,34].

For the convective heat boundary condition in heat transfer analysis, researchers have been motivated by the applications in industry and engineering, such as the solidification of cistinf, underground electric cables, material drying, etc. Cooling plants, gas turbines, thermal storage, etc. can all use convective heat boundary conditions. The stagnation point flow analysis over a porous stretching sheet in the presence of heat generation was reported by Malvandi et al. [35]. The MHD flow of Newtonian fluid with heat transfer analysis over a rotating region was attempted by Ayub et al. [36].

In pumping power the hybrid nano-fluid in the presence of convective heat and heat transfer effects were considered by Irandoost et al. [37]. A numerical approach for the thermophoretic and heat source impacts on micropolar fluid with a magnetic field was provided by Sharma et al. [38]. The convective heat transfer flow in micropolar fluid arrayed with microgrooves was analyzed by Hu et al. [39]. An exponential study of convective heat transfer in mono-hybrid nano-fluids was demonstrated by Vallaj et al. [40]. A convective heat transfer analysis of hybrid nano-fluids in the developing region tube was performed by Anoop et al. [41].

The above literature survey motivated us towards this study of convective heat transfer analysis for hybrid nano-fluids over a nonlinear curved surface. We considered the inclined magnetic field, thermal radiation, and slip boundary condition. Furthermore, the governing model was transformed into a nonlinear ODE system by using similarity transformation. The solution of these ODE systems was computed with the shooting method with an RK-4 numerical scheme. The effects of different physical and engineering parameters on the velocity and temperature of the fluid for both cases of nano-fluids: $Al_{2}O_{3}$/water ($Fe_{3}O_{4}-Al_{2}O_{3}/H_{2}O$) base fluid. Furthermore, the behavior of the skin friction and the local Nusselt number for the different physical parameters was studied in detail.

## 2. Mathematical Model

**Figure 1:**
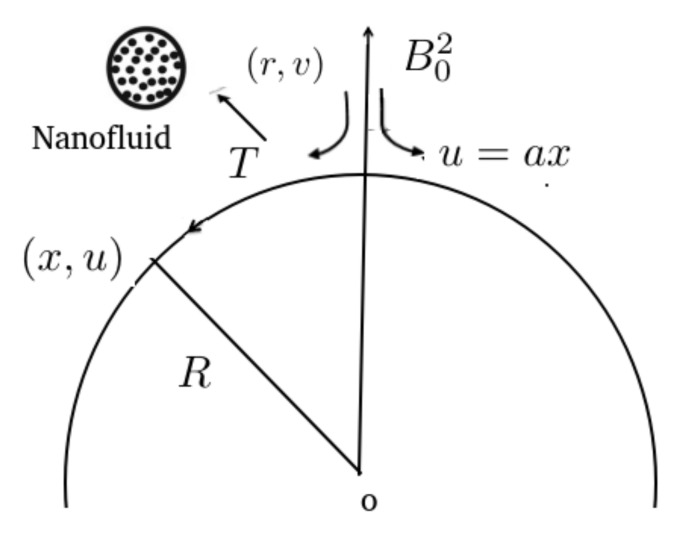
Geometry of the problem.

In this work, we considered the hybrid nano-fluid $Al_{2}O_{3}-Fe_{3}O_{4}$/water flow over a curved stretching surface. The convective heat transfer, thermal radiation, inclined magnetic field, and slip boundary condition were all taken into account. The flow was produced by a curved stretching sheet coiled with radius *r*. The stretchable surface was of a higher value than R→∞, then the surface was equal to distance *R* from the origin. The stretched surface place was along the *s*-direction with velocity component $a_{1}s^{n}=u_{w}\left(s\right)$. A magnetic field $B_{o}$ was applied at angle α lying in the range $0<\beta <\frac{\pi }{2}$ in the direction of the sheet. The geometrical interpretation of the flow problem is shown in Figure 1. The stretching curved surface of the sheet was hot by convection from a heated fluid at temperature $T_{hnf}$, which produced a convective heat transfer coefficient, $h_{hnf}$. The governing coupled partial differential equations for the above flow are given by

(1) $$\frac{\partial }{\partial r}\left((r+R)v\right)+R\frac{\partial u}{\partial s}=0$$

(2) $$\frac{u^{2}}{r+R}=\frac{1}{\rho_{hnf}}\frac{\partial p}{\partial r}$$

(3) $$\begin{array}{c} v\frac{\partial u}{\partial r}+\frac{Ru}{r+R}\frac{\partial u}{\partial s}+\frac{uv}{r+R}=-\frac{1}{\rho_{hnf}}\frac{R}{r+R}\frac{\partial p}{\partial s}-\frac{\sigma_{hnf}\sin\left(\alpha \right)B_{0}^{2}}{\rho_{hnf}}u\\ +\frac{\mu_{hnf}}{\rho_{hnf}}\left(\frac{\partial^{2}u}{\partial r^{2}}+\frac{1}{r+R}\frac{\partial u}{\partial r}-\frac{u}{{\left(r+R\right)}^{2}}\right) \end{array}$$

(4) $$v\frac{\partial T}{\partial r}\;+\frac{uR}{r+R}\frac{\partial T}{\partial s}=\frac{{\left(\rho c_{p}\right)}_{hnf}}{k_{hnf}}\left[\frac{\partial^{2}T}{\partial r}+\frac{1}{r+R}\frac{\partial \;T}{\partial r}\right]$$

The corresponding boundary conditions are

(5) $$\begin{array}{c} u=bs+L_{1}\left(\frac{\partial u}{\partial r}-\frac{u}{r+R}\right)=u_{w},v=0,k_{hnf}\frac{\partial T}{\partial r}=h\left(T_{f}-T\right),u\to 0\\ ,\frac{\partial u}{\partial r}\to 0,T\to T_{\infty }\;\mathrm{as}\;r\to \infty . \end{array}$$

Here the above expressions (v,u) represent the components in the (r,s) direction, respectively, *p* represents the pressure, *a* is the stretching rate constant, $L_{1}$ represents the slip parameter, *T* is the temperature, $k_{hnf}$ stands for the thermal conductivity of the hybrid nano-fluid, $\alpha_{hnf}=k_{hnf}/{\left(\rho c\right)}_{f}$ represents the thermal diffusivity, and *h* denotes the convective heat transfer coefficient. The effective hybrid nano-fluid density $\rho_{hnf}$ and heat capacity ${\left(\rho c_{p}\right)}_{hnf}$ may be found in [19]. These are defined as

(6) $$\begin{array}{c} C_{1}=\frac{\mu_{hnf}}{\times \mu_{f}}=\left(1-\phi_{1}\right){}^{2.5}\left(1-\phi_{2}\right){}^{2.5},\\ C_{2}=\frac{\rho_{hnf}}{\rho_{f}}=\left(1-\phi_{2}\right)\left(\frac{\phi_{1}\rho_{s_{1}}}{\rho_{f}}+\left(1-\phi_{1}\right)\right)+\frac{\phi_{2}\rho_{s_{2}}}{\rho_{f}}\\ C_{3}=\frac{{\left(\rho Cp\right)}_{hnf}}{{\left(\rho Cp\right)}_{f}}=\left(1-\phi_{2}\right)\frac{\phi_{1}\rho C_{p_{s_{1}}}}{\rho C_{p_{f}}}+\left(1-\phi_{1}\right)+\frac{\phi_{2}\rho C_{p_{s_{2}}}}{\rho C_{p_{f}}}\\ A_{3}=\frac{k_{nf}}{k_{f}}=\frac{\left(2k_{f}+k_{s_{1}}\right)-2\phi_{1}\left(k_{f}-k_{s_{1}}\right)}{\phi_{1}\left(k_{f}-k_{s_{1}}\right)+\left(2k_{f}+k_{s_{1}}\right)}\\ A_{4}=\frac{k_{nf}}{k_{f}}=\frac{\left(2A_{3}+k_{s_{2}}\right)-2\phi_{2}\left(A_{3}-k_{s_{2}}\right)}{\phi_{2}\left(A_{3}-k_{s_{2}}\right)+\left(2A_{3}+k_{s_{2}}\right)}\\ B_{1}=\frac{\left(2\sigma_{f}+\sigma_{s_{1}}\right)-2\phi_{1}\left(\sigma_{f}-\sigma_{s_{1}}\right)}{\phi_{1}\left(\sigma_{f}-\sigma_{s_{1}}\right)+\left(2\sigma_{f}+\sigma_{s_{1}}\right)}\\ B_{2}=\frac{\left(2B_{1}+\sigma_{s_{2}}\right)-2\phi_{2}\left(B_{1}-\sigma_{s_{2}}\right)}{\phi_{2}\left(B_{1}-\sigma_{s_{2}}\right)+\left(2B_{1}+\sigma_{s_{2}}\right)}. \end{array}$$

The similarity transformations are defined as

(7) $$u=bsf^{\prime }\left(\eta \right),v=\frac{-R}{r+R}\sqrt{b\upsilon }f\left(\eta \right),\xi =\sqrt{\frac{b}{\upsilon }}R,\eta =\sqrt{\frac{b}{\upsilon }}r,p=\rho b^{2}s^{2}P\left(\eta \right),\theta \left(\eta \right)=\frac{T-T_{\infty }}{T_{w}-T_{\infty }}.$$

Clearly, this satisfies the continuity equation, and Equations (1)–(4) can be written as

(8) $$\frac{\partial P}{\partial \eta }=C_{2}\frac{{\left(f^{\prime }\right)}^{2}}{\eta +\xi }$$

(9) $$\frac{1}{C_{2}}\frac{2\xi }{\eta +\xi }P\left(\eta \right)=\frac{1}{C_{2}C_{1}}\left(f^{\prime \prime \prime }+\frac{f^{\prime \prime }}{\eta +\xi }-\frac{f^{\prime }}{{\left(\eta +\xi \right)}^{2}}\right)-\frac{B_{2}}{C_{1}}\sin^{2}\alpha M^{2}f^{\prime }-\frac{k}{\eta +\xi }{\left((f^{\prime }\right)}^{2}-ff^{\prime \prime }-\frac{f^{\prime }f}{\eta +\xi })$$

(10) $$\theta^{\prime \prime }+\frac{\theta^{\prime }}{\xi +\eta }+Pr\frac{C_{3}}{A_{4}+R}f\theta^{\prime }\frac{k}{\xi +\eta }=0.$$

The corresponding boundary conditions are given by

(11) $$\begin{array}{c} f\left(\eta \right)=0,\;f^{\prime }\left(\eta \right)=1+\frac{K_{1}}{\xi }\left(\xi f^{\prime \prime }\left(\eta \right)-f^{\prime }\left(\eta \right)\right),\;\theta^{\prime }\left(\eta \right)=-\frac{\left(1-\theta (\eta )\right)}{A_{4}}\;\mathrm{at}\;\eta \to 0,\\ f^{\prime }\left(\eta \right)=0,f^{\prime \prime }\left(\eta \right)=0,\theta \left(\eta \right)=0\;\mathrm{at}\;\eta \to \infty . \end{array}$$

In the above formulation, *f* and θ represent the velocity and temperature, and ξ, $R,M,A_{1},Pr$, and $K_{1}$ are the curvature, magnetic value, thermal radiation, Biot number, Prandtl number, and velocity slip parameter, respectively, and are given as follows:

$$\eta =\xi \sqrt{b\upsilon },R=\frac{4\sigma^{*}T_{\infty }^{3}}{3k^{*}\rho c_{p}},M=\frac{\sigma B_{0}^{2}}{b\rho_{f}},\;A_{1}=\frac{k}{k_{f}}\sqrt{\frac{V_{f}}{b}},Pr=\frac{v_{f}Cp}{k_{f}},K_{1}=L_{1}\sqrt{\frac{b}{\nu_{f}}}$$

.

By eliminating the pressure term form Equations (8) and (9), we obtained

(12) $$\begin{array}{c} f^{⁗}+\frac{f^{\prime }}{{\left(\xi +\eta \right)}^{3}}+C_{2}C_{1}\left(-\frac{kff^{\prime }}{{\left(\xi +\eta \right)}^{3}}+\frac{k\left(ff^{\prime \prime }-f^{\prime 2}\right)}{{\left(\xi +\eta \right)}^{2}}+\frac{\xi \left(f^{\prime \prime \prime }f^{\prime }-ff^{\prime \prime }\right)}{\xi +\eta }\right)\\ +B_{1}C_{1}M^{2}\sin\left(\alpha \right)\left(\frac{f^{\prime }}{\xi +\eta }+f^{\prime \prime }\right)+\frac{2f^{\prime \prime \prime }}{\xi +\eta }-\frac{f^{\prime \prime }}{{\left(\xi +\eta \right)}^{2}}=0. \end{array}$$

The corresponding boundary conditions are

(13) $$f\left(0\right)=0,\;f^{\prime }\left(\eta \right)=1+\frac{K_{1}}{\xi }\left(\xi f^{\prime \prime }\left(\eta \right)-f^{\prime }\left(\eta \right)\right)\mathrm{at}\;\eta =0,\;f^{\prime }\left(\eta \right)=0,f^{\prime \prime }\left(\eta \right)=0,\mathrm{at}\;\eta \to \infty .$$

We determined the pressure thus:

(14) $$\begin{array}{c} P=C_{2}\left(\frac{ff^{\prime }}{2(\xi +\eta )}+\frac{1}{2}ff^{\prime \prime }-\frac{1}{2}f^{\prime 2}\right)+\frac{1}{C_{1}}\left(\frac{\xi +\eta }{2\xi }f^{\prime \prime \prime }+\frac{1}{2\xi }f^{\prime \prime }-\frac{f^{\prime }}{2\xi (\xi +\eta )}\right)\\ -B_{1}M^{2}\sin\left(\alpha \right)f^{\prime }\left(\frac{\xi +\eta }{2\xi }\right). \end{array}$$

Here $q_{m}$ is the heat transfer, and $\tau_{w}$ is the wall shear stress, given as

(15) $$\tau_{w}=\mu_{hnf}{\left[\frac{\partial u}{\partial r}-\frac{u}{r+R}\right]}_{r=0}\;q_{w}=-{\left[k_{hnf}\frac{\partial \hat{T}}{\partial r}+\frac{\partial q_{r}}{\partial r}\right]}_{r=0}.$$

The dimensionless form of Equation (15) can be formulated as

(16) $$\begin{array}{cc} C_{f}\sqrt{Re_{x}} & =\frac{1}{C1}\left(f^{\prime \prime }\left(0\right)-\frac{f^{\prime }\left(0\right)}{\xi }\right),\\ \frac{Nu_{x}}{\sqrt{Re_{x}}} & =-\left[A_{3}+R_{d}\right]\theta^{\prime }(0) \end{array}$$

Where $C_{f}\sqrt{Re_{x}}$ and $\frac{Nu_{x}}{\sqrt{Re_{x}}}$ represent the skin fraction and local Nusselt number, respectively.

## 3. Shooting Method

The shooting method is a numerical approach generally used for the solution of the BVP by reducing it to the system of an initial value problem. Equations (10)–(12) are the system of non-linear coupled ODEs of order four in f(ξ) and order two in θ(η), respectively. Rearranging Equations (10)–(12) with boundary conditions will take the form of

(17) $$\begin{array}{c} f^{⁗}=-\frac{f^{\prime }}{{\left(\xi +\eta \right)}^{3}}-C_{2}C_{1}\left(-\frac{kff^{\prime }}{{\left(\xi +\eta \right)}^{3}}+\frac{k\left(ff^{\prime \prime }-f^{\prime 2}\right)}{{\left(\xi +\eta \right)}^{2}}+\frac{\xi \left(f^{\prime \prime \prime }f^{\prime }-ff^{\prime \prime }\right)}{\xi +\eta }\right)\\ -B_{1}C_{1}M^{2}\sin\left(\alpha \right)\left(\frac{f^{\prime }}{\xi +\eta }+f^{\prime \prime }\right)-\frac{2f^{\prime \prime \prime }}{\xi +\eta }+\frac{f^{\prime \prime }}{{\left(\xi +\eta \right)}^{2}}, \end{array}$$

(18) $$\begin{array}{c} \theta^{\prime \prime }=-\frac{\theta^{\prime }}{\xi +\eta }-Pr\frac{C_{3}}{A_{4}+R}f\theta^{\prime }\frac{k}{\xi +\eta }. \end{array}$$

To reduce the higher-order nonlinear coupled ODEs into a first-order ODE system, let us consider

(19) $$\begin{array}{cc} & f=u_{1},f^{\prime }=u_{2},\;f^{\prime \prime }=u_{3},\;f^{\prime \prime \prime }=u_{4}\mathrm{and}u_{4}^{\prime }=f^{⁗}, \end{array}$$

(20) $$\begin{array}{cc} \; & \theta =u_{5},\;\theta^{\prime }=u_{6}\;\mathrm{and}\;\theta^{\prime \prime }=u_{7}^{\prime }. \end{array}$$

The nonlinear coupled ODE system is reduced into a first-order ODE system. It can be defined with the new variables as

(21) $$\begin{array}{cc} & u_{1}^{\prime }=u_{2},\\ \; & u_{2}^{\prime }=u_{3},\\ \; & u_{3}^{\prime }=u_{4}\\ \; & u_{4}^{\prime }=-\frac{u_{2}}{{\left(\xi +\eta \right)}^{3}}-C_{2}C_{1}\left\{-\frac{ku_{1}u_{2}}{{\left(\xi +\eta \right)}^{3}}+\frac{k\left(u_{1}u_{3}-u_{2}^{2}\right)}{{\left(\xi +\eta \right)}^{2}}+\frac{\xi \left(u_{4}u_{1}-u_{1}u_{3}\right)}{\xi +\eta }\right\}\\ & -B_{1}C_{1}M^{2}\sin\left(\alpha \right)\left(\frac{u_{2}}{\xi +\eta }+u_{3}\right)-\frac{2u_{4}}{\xi +\eta }+\frac{u_{3}}{{\left(\xi +\eta \right)}^{2}}\\ \; & u_{5}^{\prime }=u_{6}\\ & u_{6}^{\prime }=-\frac{u_{6}}{\xi +\eta }-Pr\frac{C_{3}}{A_{4}+R}u_{1}u_{6}\frac{k}{\xi +\eta }. \end{array}$$

The corresponding boundary conditions are

(22) $$\begin{array}{cc} & u_{1}\left(0\right)=0,u_{2}\left(0\right)=1+K_{1}\left(u_{3}\left(0\right)-\frac{u_{2}\left(0\right)}{\xi }\right)\;u_{6}\left(0\right)=-A_{1}\left(\frac{1-u_{4}\left(0\right)}{A_{4}}\right). \end{array}$$

The next task is to solve the above seven first-order ODE systems (20)–(21) via a shooting method with RK-4. For any numerical solution this evidently requires seven initial guesses, whereas four initial guesses were given and the other three initial guesses were $u_{2}\left(\eta \right)$, $u_{4}\left(\eta \right)$, and $u_{6}\left(\eta \right)$. These were defined as η→∞. Hence, it was considered that $\left(u_{2}\left(0\right),u_{4}\left(0\right),u_{6}\left(0\right)\right)=\left(q_{1},q_{2},q_{3}\right)$. These unknown required three initial guesses $(u_{2}\left(0\right)$, $u_{4}\left(0\right)$, and $u_{6}\left(0\right))$ were computed by the Newton iterative scheme. The main step of this numerical solution is to select the suitable finite values for boundary conditions. The step size and convergence criteria were taken to be ▵η=0.02 and $TOL=10^{-5}$, respectively, for our numerical solution.

## 4. Results and Discussions

First of all, we computed the solution of the resulting ODE system given in Equations (10)–(12) with a shooting method along with RK-4 Method. For the numerical computations we used the specific values for the parameters given in Table 1. Further, We compared the present work with previously published data and found a good agreement between them. The comparison is given in Table 2. The characteristics of different physical parameters, namely temperature and velocity, for the hybrid nano-fluid ($Al_{2}O_{3}$/water and $Fe_{3}O_{4}-Al_{2}O_{3}/H_{2}O$) were determined and presented.

**Figure 2:**
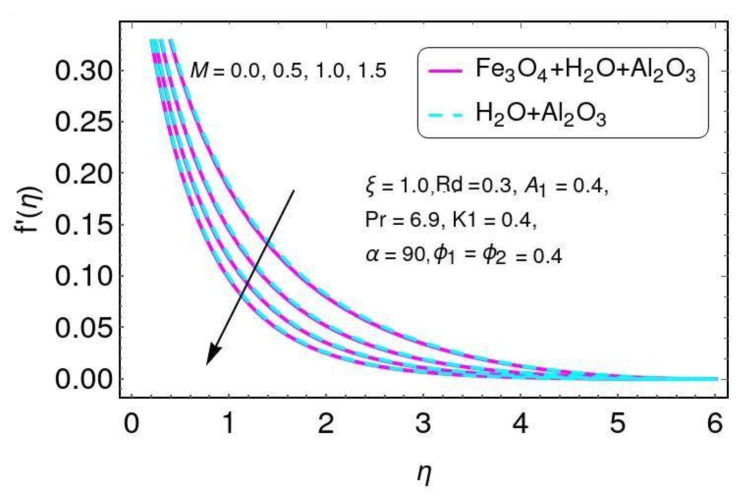
Characteristics of *M* for the velocity distribution $f^{\prime }$.

**Figure 3:**
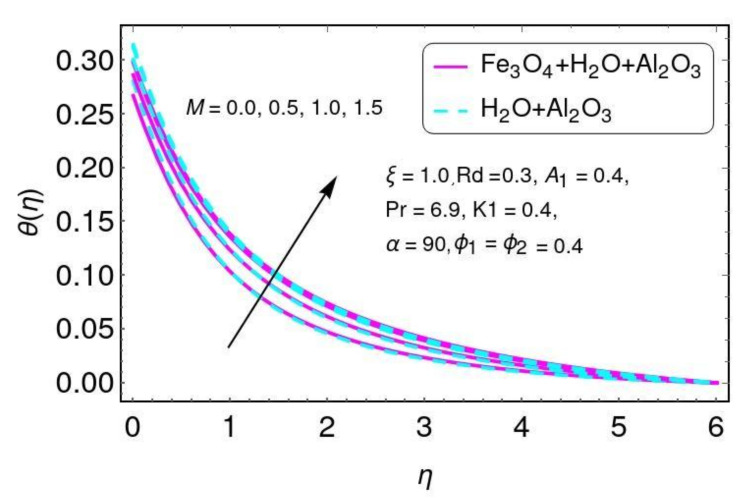
The result of *M* on θ(η).

**Figure 4:**
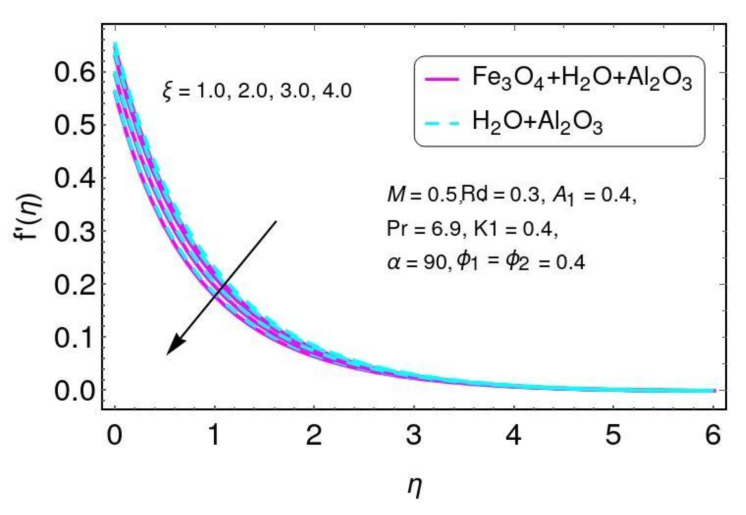
The distribution of $f^{\prime }\left(\eta \right)$ for ξ.

**Figure 5:**
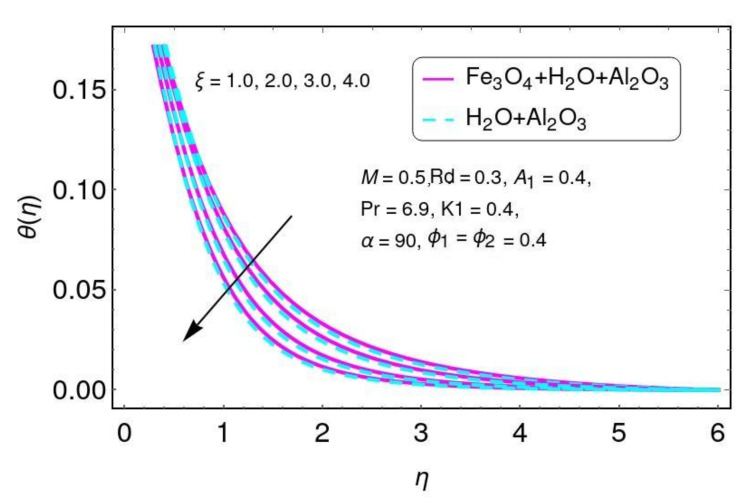
Temperature field for ξ.

The effect of the magnetic parameter on velocity is shown in Figure 2 for the hybrid nano-fluid ($Al_{2}O_{3}$/Water and $Fe_{3}O_{4}-Al_{2}O_{3}/H_{2}O$). A Lorentz force was created due to the existence of a magnetic field, which enhances the interaction in electrically conducting the fluid motion and, therefore, reduces the fluid motion. Here it is noticed that the fluid temperature rises by greater values of *M*, as shown in Figure 3 for the hybrid nano-fluid ($Al_{2}O_{3}$/water and $Fe_{3}O_{4}-Al_{2}O_{3}/H_{2}O$). The effect of the curvature parameter ξ on velocity is plotted in Figure 4 for the hybrid nano-fluid ($Al_{2}O_{3}$/Water and $Fe_{3}O_{4}-Al_{2}O_{3}/H_{2}O$). The velocity rises by higher values of ξ for both $Al_{2}O_{3}/H_{2}O$ and $Fe_{3}O_{4}-Al_{2}O_{3}/H_{2}O$ nanomaterials. The higher values of ξ mean that the radius of the sheet is enhanced, which increases the fluid motion. Similarly, the same result for the curvature parameter ξ is shown in Figure 5 in the temperature field for $Al_{2}O_{3}/H_{2}O$ and the hybrid nano-fluid ($Fe_{3}O_{4}-Al_{2}O_{3}/H_{2}O$).

**Figure 6:**
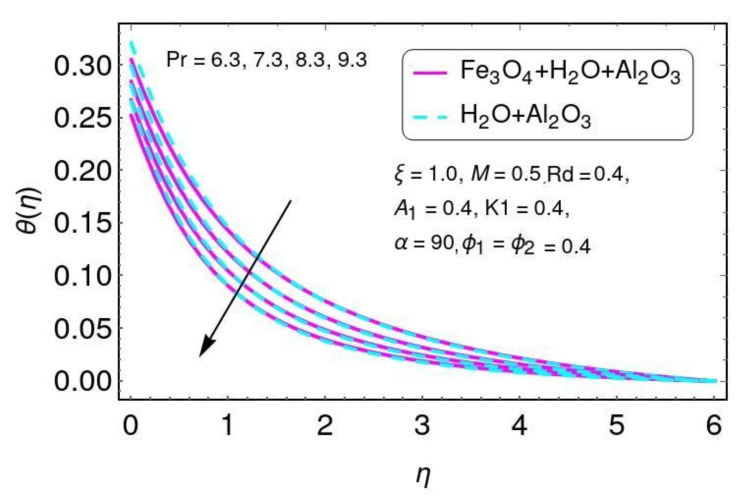
The gradient of (η) for Pr.

**Figure 7:**
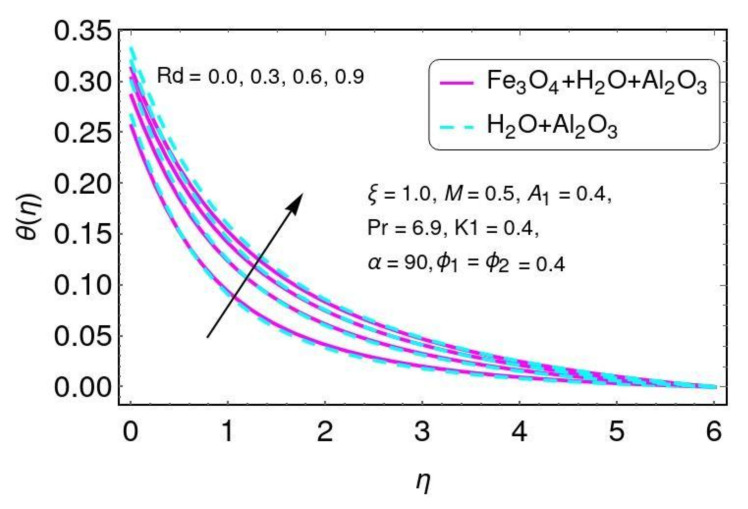
The impact of *R* for θ(η).

Figure 6 is plotted for the temperature field with different values of Prandtl number Pr for the hybrid nano-fluid ($Al_{2}O_{3}/H_{2}O$ and $Fe_{3}O_{4}-Al_{2}O_{3}/H_{2}O$). Here a reduction in the temperature was found with higher values of Pr for both $Al_{2}O_{3}/H_{2}O$ and $Fe_{3}O_{4}-Al_{2}O_{3}/H_{2}O$ nanomaterials. The effects of the thermal radiation parameter Rd on temperature can be seen in Figure 7 for hybrid nano-fluid ($Al_{2}O_{3}/H_{2}O$ and $Fe_{3}O_{4}-Al_{2}O_{3}/H_{2}O$) flow. The temperature was increased by increasing the radiation parameter for both nanomaterials ($Al_{2}O_{3}/H_{2}O$ and $Fe_{3}O_{4}-Al_{2}O_{3}/H_{2}O$). This increase occurs due to the increase in temperature $T_{\infty }^{3}$.

**Figure 8:**
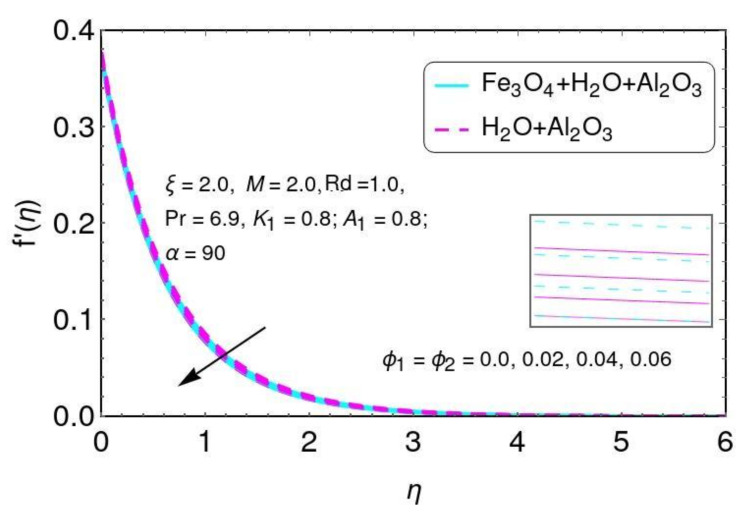
The distribution of $f^{\prime }\left(\eta \right)$ for $\phi_{1},\phi_{2}$.

**Figure 9:**
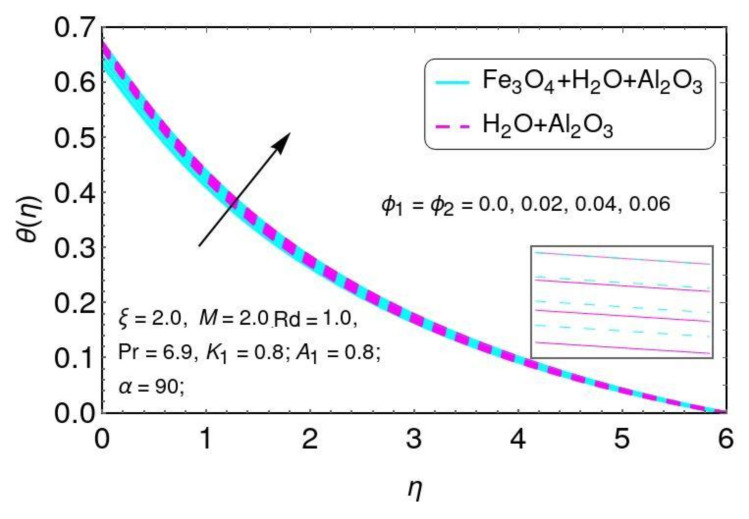
The characteristics of $\phi_{1},\phi_{2}$ on the temperature field.

The influence of velocity $f^{\prime }\left(\eta \right)$ is presented in Figure 8 by volume fractions $\phi_{1},\phi_{2}$. It is seen that the velocity effect $f^{\prime }\left(\eta \right)$ declined by greater values of the nano-particle volume fractions $\phi_{1},\phi_{2}$ for the hybrid nano-fluids. Physically, the higher values of the nano-particle volume fraction of $Al_{2}O_{3}$/water and $Al_{2}O_{3}+Fe_{3}O_{4}/$water were due to the thickness of the momentum boundary layer. The influence of fluid temperature θ(η) is presented in Figure 9 by nano-particle volume fractions $\phi_{1},\phi_{2}$.

**Figure 10:**
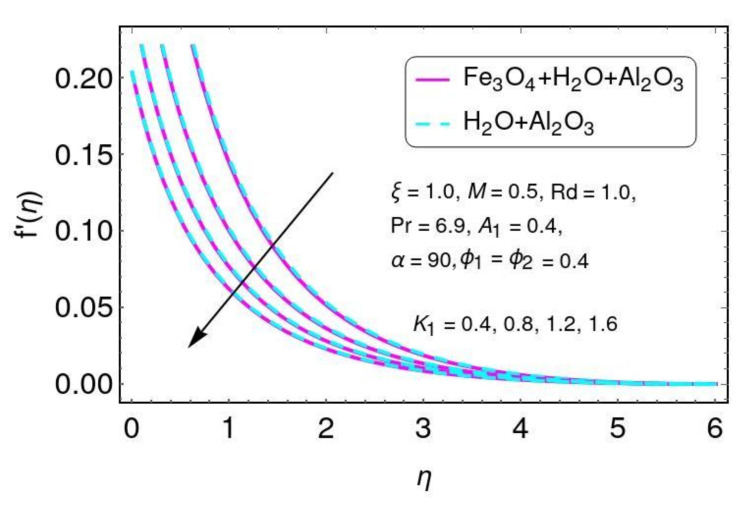
The distribution of $f^{\prime }\left(\eta \right)$ for $K_{1}$.

It is clearly seen that the fluid temperature θ(η) increased by greater values of the nano-particle volume fraction $\phi_{1},\phi_{2}$ for the hybrid nano-fluids. In fact, the higher values of thermal conduction were improved by the greater thermal diffusivity. The fluid temperature θ() field is directly related to both ($\phi_{1},\phi_{2}$), resulting in increases in the fluid temperature for $Al_{2}O_{3}$/Water and $Al_{2}O_{3}+Fe_{3}O_{4}/$water. The velocity behavior is plotted in Figure 10 for higher values of the velocity slip parameter for both $Al_{2}O_{3}/H_{2}O$ and $Fe_{3}O_{4}-Al_{2}O_{3}/H_{2}O$ nano-particles. It is noticed that velocity decreases with greater values of $K_{1}$ for both $Al_{2}O_{3}/H_{2}O$ and $Fe_{3}O_{4}-Al_{2}O_{3}/H_{2}O$ nano-particles.

**Figure 11:**
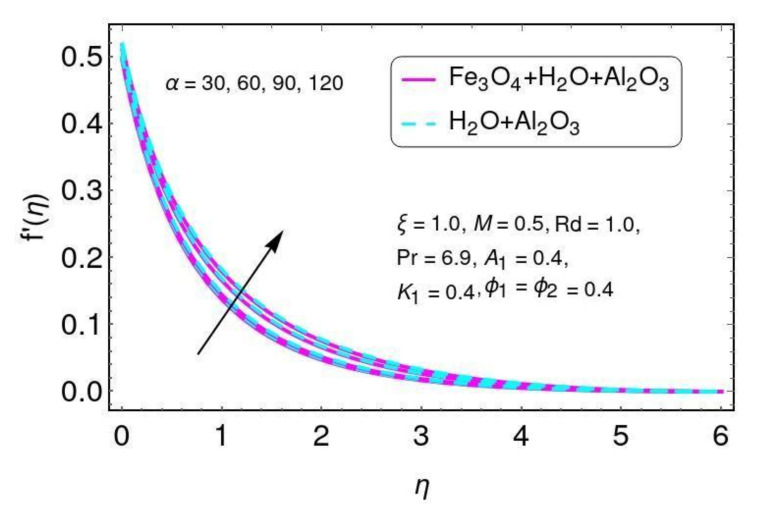
The distribution of $f^{\prime }\left(\eta \right)$ for α.

**Figure 12:**
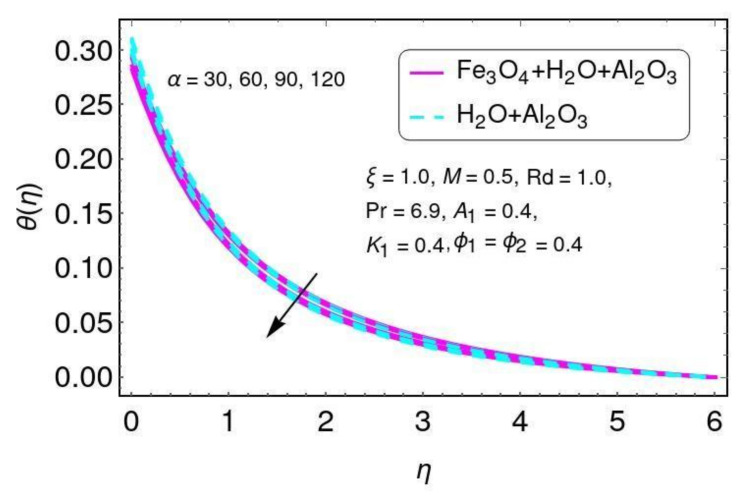
The characteristics of α on the temperature field.

Figure 11 shows the velocity for angle of inclination α for both $Al_{2}O_{3}/H_{2}O$ and $Fe_{3}O_{4}-Al_{2}O_{3}/H_{2}O$ nanomaterials. Here it is noticed that the velocity was reduced with larger values of angle of inclination α. This is because by increasing the angle of inclination, the effect of the magnetic field on fluid particles increases, which enhances the Lorentz force. Figure 12 shows the angle of inclination effects on temperature for the hybrid nano-fluids $Al_{2}O_{3}/H_{2}O$ and $Fe_{3}O_{4}-Al_{2}O_{3}/H_{2}O$. Here it is seen that the temperature increased with larger values of α for both nano-particles, $Al_{2}O_{3}/H_{2}O$ and $Fe_{3}O_{4}-Al_{2}O_{3}/H_{2}O$. The higher angle values correspond to a larger magnetic field which opposes the fluid motion. Hence, this increases the value of the temperature.

**Figure 13:**
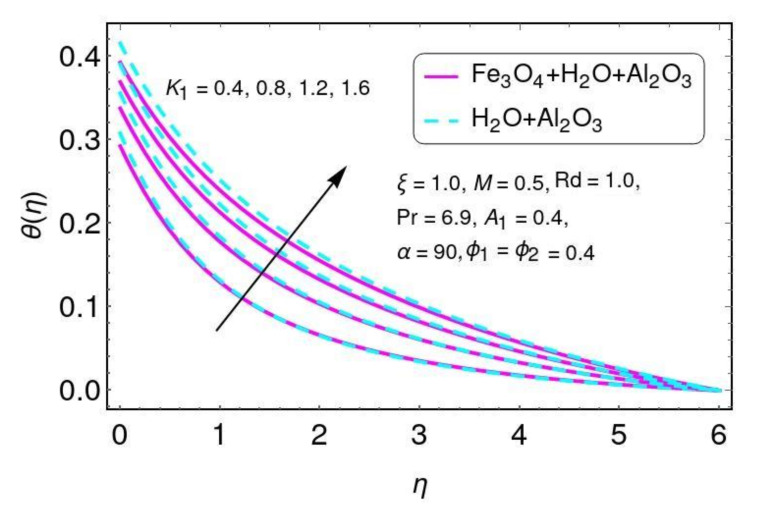
The properties of $K_{1}$ on the temperature field.

**Figure 14:**
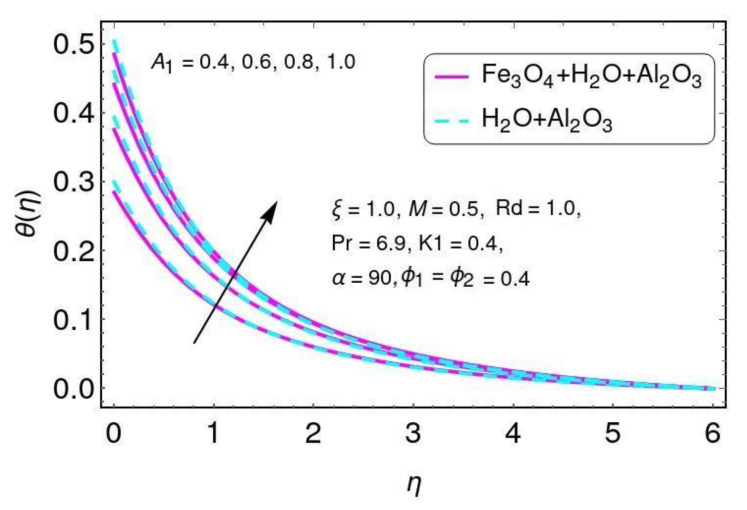
The properties of $A_{1}$ on the temperature field.

The effects of the velocity slip parameter $K_{1}$ on temperature field $\theta (\eta_{)}$ are found in Figure 13. It is noticed that temperature increased for greater values of $K_{1}$ and also increased the thermal boundary layer for both hybrid nano-fluids considered in this work. The impacts of convective heat transfer are shown in Figure 14 for the temperature field. It is concluded that the temperature rises for hybrid nano-particles $Al_{2}O_{3}/H_{2}O$ and $Fe_{3}O_{4}-Al_{2}O_{3}/H_{2}O$ with higher values of convective heat transfer $A_{1}$.

Table 3 displays the numerical properties of Nusselt numbers and skin fractions for different physical parameters. The skin friction decreased with higher values of *M* and $\phi_{2}$ and increased with higher values of *R* and ξ. The Nusselt number increased with higher values of Lt and $\phi_{2}$ and decreased with higher values of *M*, ξ, *R*, and Pr.

## 5. Conclusions

In this work, the effects of velocity slip on a hybrid nano-fluid over a curved stretching sheet were considered. Furthermore, ($Al_{2}O_{3}$) aluminum oxide and ferro ($Fe_{3}O_{4}$) nano-particles, thermal radiation, and convective heat transfer were examined. We transformed the governing model into a non-linear coupled ODE system by using similarity transformation. Then, the solution of these ODEs was computed by the shooting method with the RK-method of order four. The characteristics of temperature and velocity profiles for the physical parameters involved were presented graphically. Moreover, the heat transfer rate and skin friction coefficients were displayed in tables for both nano-fluids and hybrid nano-fluids. We draw the following conclusions:

- There was a reduction in the velocity $f^{\prime }\left(0\right)$ with increasing the values of ξ, $\phi_{2}=\phi_{1}$, $K_{1}$, and *M* for aluminum oxide ($Al_{2}O_{3}$) and ferro ($Fe_{3}O_{4}$) nano-particles.
- There was an increase in temperature gradients θ(η) with higher values of *R* for aluminum oxide ($Al_{2}O_{3}$) and ferro ($Fe_{3}O_{4}$) nano-particles.
- There was a decrease in the temperature gradients θ(η) with higher values of Pr aluminum oxide ($Al_{2}O_{3}$) and ferro ($Fe_{3}O_{4}$) nano-particles.

## Figures and Tables

**Table 1:** Thermo-physical aspects of different fluids and nano-fluids.

Properties	$H_{2}O$/Water	${\mathrm{Al}}_{2}O_{3}$ Aluminum Oxide	${\mathrm{Fe}}_{3}O_{4}$ Ferrofluid
ρ (kg/m${}^{3}$)	997.1	3970	5180
Cp (JK${}^{-1}$/kg)	4180.0	765.0	650
*k* (W m${}^{-1}$/K)	0.6071	40.0	9.7
σ (s/m)	0.05	3.5×$\;10^{7}$	0.74×$\;10^{7}$

**Table 2:** Comparison of the current results with those from previous published research with different values of Pr by taking $Lt=\phi_{1}=\phi_{2}=K_{1}=A_{1}=0$.

Pr	Saba et al. [42]	Grubkal et al. [43]	Ishak et al. [44]	Present Work
0.72	0.8086	0.8086	0.8058	0.8086
1.0	1.0000	1.0000	1.0000	1.0000
3.0	1.9237	1.9237	1.9237	1.9237

**Table 3:** The variation of the skin friction coefficient and Nusselt number for both the nano-fluid and the hybrid nano-fluid.

$\phi_{2}$	M	R	Lt	Pr	ξ	Skin Friction Co-Efficient, $-f^{\prime \prime }\left(0\right)$		Nusselt Number $-\theta^{\prime }\left(0\right)$	
						** $Fe_{3}O_{4}-Al_{2}O_{3}$ **	** $Al_{2}O_{3}$ **	$Fe_{3}O_{4}-Al_{2}O_{3}$	$Al_{2}O_{3}$
0.005	2.0	1.0	0.4	6.2	0.3	5.04486	5.4346	0.380146	0.379330
0.02						5.05593	5.44837	0.365899	0.365116
0.04						5.06784	5.46335	0.347978	0.347237
0.04	0.0	1.0	0.4	6.2	0.3	5.34173	5.75861	0.328696	0.327995
	0.2					5.34159	5.75840	0.326459	0.325826
	0.4					5.3415	5.75825	0.324735	0.324156
	0.6					5.34144	5.75814	0.323365	0.322833
0.04	0.2	0.0	0.4	6.2	0.3	3.20277	3.20512	0.334256	0.334621
		1.0				3.43466	3.50167	0.332969	0.332993
		2.0				3.97763	4.15909	0.330530	0.330179
		3.0				4.63385	4.93133	0.328304	0.327762
0.04	0.2	4.0	0.3	6.2	0.3	5.34159	5.75840	0.326459	0.325826
			0.6			4.21659	4.65584	0.310688	0.309276
			0.9			3.88472	4.32912	0.304040	0.302058
			1.2			3.73050	4.17672	0.300530	0.298137
0.04	2.0	1.0	0.4	6.2	0.3	5.34159	5.75840	0.326459	0.325826
				7.0		5.34169	5.75855	0.328092	0.327410
				8.0		5.34182	5.75875	0.330049	0.329312
				9.0		5.34196	5.75898	0.331916	0.331131
0.04	2.0	1.0	0.4	6.2	0.4	5.34159	5.75840	0.326459	0.325826
					0.6	5.33985	5.75631	0.448490	0.447358
					0.8	5.33904	5.75476	0.551573	0.549915
					1.0	5.33835	5.75376	0.639806	0.637616

## Data Availability

Data sharing is not applicable to this article as no data sets were generated nor analyzed during the current study.

## Nomenclature

MHD	Magnetohydrodynamics	RK	Runge-Kutta
IVP	Initial value problem	BVP	Boundary value problem
*M*	Magnetic parameter (Wb/m${}^{2}$)	$\mu_{o}$	Magnetic permeability
Pr	Prandtl number	Γ, *b*	Rate constant of fluid material
			parameter
$C_{f}$	Local skin friction	$K_{1}$	Slip boundary condition
$C_{p}$	Specific heat m${}^{2}$ s${}^{-2}$	*f*	Dimensionless velocity
$q_{w}$	Wall heat flux	ζ	Stefan-Boltzmann constant
$Nu_{x}$	Nusselt number	*b*	Stretching rate constant
*r*	Radius	$\nu_{hnf}$	Kinematic viscosity m${}^{2}$s${}^{-1}$
$Re_{x}$	Reynolds number	$A_{1}$	Convective heat transfer
$T_{w}$	Wall temperature (K)	Rd	Radiation parameter (Rad)
*u*	Velocity along *x*-axis	ξ	Curvature parameter
$\mu_{hnf}$	Dynamic viscosity (kg/ms)	$u_{w}$	Stretching velocity along *x*-axis
$T_{\infty }$	Ambient temperature (K)	*p*	Pressure
$\phi_{1},\phi_{2}$	Volume fraction of nano-particles	α	Inclination parameter

## References

[1] Crane L.J. (1970). Flow past a stretching plate. Z. Angew. Math. Phys. ZAMP.

[2] Vleggaar J. (1977). Laminar boundary-layer behaviour on continuous, accelerating surfaces. Chem. Eng. Sci..

[3] Wang C. (1984). The three-dimensional flow due to a stretching flat surface. Phys. Fluids.

[4] Sajid M., Ali N., Javed T., Abbas Z. (2010). Stretching a curved surface in a viscous fluid. Chin. Phys. Lett..

[5] Roşca N.C., Pop I. (2015). Unsteady boundary layer flow over a permeable curved stretching/shrinking surface. Eur. J. Mech.-B/Fluids.

[6] Saif R.S., Muhammad T., Sadia H., Ellahi R. (2020). Boundary layer flow due to a nonlinear stretching curved surface with convective boundary condition and homogeneous-heterogeneous reactions. Phys. A Stat. Mech. Its Appl..

[7] Murtaza M., Tzirtzilakis E., Ferdows M. (2018). A Note on MHD Flow and Heat Transfer over a Curved Stretching Sheet by Considering Variable Thermal Conductivity. Int. J. Math. Comput. Sci..

[8] Shi Q.H., Shabbir T., Mushtaq M., Khan M.I., Shah Z., Kumam P. (2021). Modelling and numerical computation for flow of micropolar fluid towards an exponential curved surface: A Keller box method. Sci. Rep..

[9] Naveed M., Abbas Z., Sajid M. (2016). MHD flow of micropolar fluid due to a curved stretching sheet with thermal radiation. J. Appl. Fluid Mech..

[10] Choi S.U., Eastman J.A. (1995). Enhancing Thermal Conductivity of Fluids with Nanoparticles.

[11] Oztop H.F., Abu-Nada E. (2008). Numerical study of natural convection in partially heated rectangular enclosures filled with nanofluids. Int. J. Heat Fluid Flow.

[12] Li Z., Xia D., Zhou X., Cao J., Chen W., Wang X. (2021). The hydrodynamics of self-rolling locomotion driven by the flexible pectoral fins of 3-D bionic dolphin. J. Ocean. Eng. Sci..

[13] Gyergyek S., Kocjan A., Bjelić A., Grilc M., Likozar B., Makovec D. (2018). Magnetically separable Ru-based nano-catalyst for the hydrogenation/hydro-deoxygenation of lignin-derived platform chemicals. Mater. Res. Lett..

[14] Sheremet M.A., Mehryan S., Kashkooli F.M., Pop I., Ghalambaz M. (2019). Local thermal non-equilibrium analysis of conjugate free convection within a porous enclosure occupied with Ag–MgO hybrid nanofluid. J. Therm. Anal. Calorimetry.

[15] Zekavatmand S.M., Rezazadeh H., Inc M., Vahidi J., Ghaemi M.B. (2021). The new soliton solutions for long and short-wave interaction system. J. Ocean. Eng. Sci..

[16] Kashani D.A., Dinarvand S., Pop I., Hayat T. (2019). Effects of dissolved solute on unsteady double-diffusive mixed convective flow of a Buongiorno’s two-component nonhomogeneous nanofluid. Int. J. Numer. Methods Heat Fluid Flow.

[17] Suresh S., Venkitaraj K., Selvakumar P., Chandrasekar M. (2011). Synthesis of Al_2_O_3_–Cu/water hybrid nanofluids using two step method and its thermo physical properties. Colloids Surf. A Physicochem. Eng. Asp..

[18] Shoaib M., Raja M.A.Z., Sabir M.T., Awais M., Islam S., Shah Z., Kumam P. (2021). Numerical analysis of 3-D MHD hybrid nanofluid over a rotational disk in presence of thermal radiation with Joule heating and viscous dissipation effects using Lobatto IIIA technique. Alex. Eng. J..

[19] Devi S.S.U., Devi S.A. (2016). Numerical investigation of three-dimensional hybrid Cu–Al_2_O_3_/water nanofluid flow over a stretching sheet with effecting Lorentz force subject to Newtonian heating. Can. J. Phys..

[20] Hassan M., Marin M., Ellahi R., Alamri S.Z. (2018). Exploration of convective heat transfer and flow characteristics synthesis by Cu–Ag/water hybrid-nanofluids. Heat Transf. Res..

[21] Dinarvand S. (2019). Nodal/saddle stagnation-point boundary layer flow of CuO–Ag/water hybrid nanofluid: A novel hybridity model. Microsyst. Technol..

[22] Saeed A., Alghamdi W., Mukhtar S., Shah S.I.A., Kumam P., Gul T., Nasir S., Kumam W. (2021). Darcy-Forchheimer hybrid nanofluid flow over a stretching curved surface with heat and mass transfer. PLoS ONE.

[23] Gohar T.S.K., Khan I., Gul T., Bilal M. (2022). Mixed convection and thermally radiative hybrid nanofluid flow over a curved surface. Nanofluids Therm. Appl..

[24] Waini I., Ishak A., Pop I. (2020). MHD flow and heat transfer of a hybrid nanofluid past a permeable stretching/shrinking wedge. Appl. Math. Mech..

[25] Anuar N.S., Bachok N., Arifin N.M., Rosali H. (2019). Effect of suction/injection on stagnation point flow of hybrid nanofluid over an exponentially shrinking sheet with stability analysis. CDF Lett..

[26] Ahmed A., Nadeem S. (2017). Effects of magnetohydrodynamics and hybrid nanoparticles on a micropolar fluid with 6-types of stenosis. Results Phys..

[27] Yousefi M., Dinarvand S., Yazdi M.E., Pop I. (2018). Stagnation-point flow of an aqueous titania-copper hybrid nanofluid toward a wavy cylinder. Int. J. Numer. Methods Heat Fluid Flow.

[28] Chu Y.M., Nazir U., Sohail M., Selim M.M., Lee J.R. (2021). Enhancement in thermal energy and solute particles using hybrid nanoparticles by engaging activation energy and chemical reaction over a parabolic surface via finite element approach. Fractal Fract..

[29] Bjelić A., Grilc M., Gyergyek S., Kocjan A., Makovec D., Likozar B. (2018). Catalytic hydrogenation, hydrodeoxygenation, and hydrocracking processes of a lignin monomer model compound eugenol over magnetic Ru/C–Fe_2_O_3_ and mechanistic reaction microkinetics. Catalysts.

[30] Zhao T.H., Khan M.I., Chu Y.M. (2021). Artificial neural networking (ANN) analysis for heat and entropy generation in flow of non-Newtonian fluid between two rotating disks. Math. Methods Appl. Sci..

[31] Nazeer M., Hussain F., Khan M.I., El-Zahar E.R., Chu Y.M., Malik M. (2022). Theoretical study of MHD electro-osmotically flow of third-grade fluid in micro channel. Appl. Math. Comput..

[32] Gyergyek S., Kocjan A., Grilc M., Likozar B., Hočevar B., Makovec D. (2020). A hierarchical Ru-bearing alumina/magnetic iron-oxide composite for the magnetically heated hydrogenation of furfural. Green Chem..

[33] Zhao T.H., He Z.Y., Chu Y.M. (2021). Sharp bounds for the weighted Hölder mean of the zero-balanced generalized complete elliptic integrals. Comput. Methods Funct. Theory.

[34] Chu Y.M., Shankaralingappa B., Gireesha B., Alzahrani F., Khan M.I., Khan S.U. (2022). Combined impact of Cattaneo-Christov double diffusion and radiative heat flux on bio-convective flow of Maxwell liquid configured by a stretched nano-material surface. Appl. Math. Comput..

[35] Malvandi A., Hedayati F., Nobari M. (2014). An HAM analysis of stagnation-point flow of a nanofluid over a porous stretching sheet with heat generation. J. Appl. Fluid Mech..

[36] Ayub R., Ahmad S., Asjad M.I., Ahmad M. (2021). Heat Transfer Analysis for Viscous Fluid Flow with the Newtonian Heating and Effect of Magnetic Force in a Rotating Regime. Complexity.

[37] Irandoost Shahrestani M., Houshfar E., Ashjaee M., Allahvirdizadeh P. (2021). Convective heat transfer and pumping power analysis of MWCNT+ Fe3O4/water hybrid nanofluid in a helical coiled heat exchanger with orthogonal rib turbulators. Front. Energy Res..

[38] Sharma R.P., Mishra S. (2021). A numerical simulation for the control of radiative heat energy and thermophoretic effects on MHD micropolar fluid with heat source. J. Ocean. Eng. Sci..

[39] Hu H.P. (2021). Theoretical Study of Convection Heat Transfer and Fluid Dynamics in Microchannels with Arrayed Microgrooves. Math. Probl. Eng..

[40] Vallejo J.P., Prado J.I., Lugo L. (2021). Hybrid or mono nanofluids for convective heat transfer applications. A critical review of experimental research. Appl. Therm. Eng..

[41] Anoop K., Sundararajan T., Das S.K. (2009). Effect of particle size on the convective heat transfer in nanofluid in the developing region. Int. J. Heat Mass Transf..

[42] Saba F., Ahmed N., Hussain S., Khan U., Mohyud-Din S.T., Darus M. (2018). Thermal analysis of nanofluid flow over a curved stretching surface suspended by carbon nanotubes with internal heat generation. Appl. Sci..

[43] Grubka L., Bobba K. (1985). Heat transfer characteristics of a continuous stretching surface with variable temperature. J. Heat Transf..

[44] Ishak A., Nazar R., Pop I. (2009). Boundary layer flow and heat transfer over an unsteady stretching vertical surface. Meccanica.

